# P-1322. A Comparative Study Assessing the Impact of High Dose versus Standard Dose Tigecycline on Liver Function in the Setting of Multi-Drug Resistant Gram-Negative Bacterial Infections

**DOI:** 10.1093/ofid/ofaf695.1510

**Published:** 2026-01-11

**Authors:** John Hennagin, Shivam Shah, Samantha Aguilar, Mollie VanNatta, Alexandre E Malek

**Affiliations:** Louisiana State University Health Shreveport, Shreveport, LA; Louisiana State University Health Sciences Center at Shreveport, Shreveport, Louisiana; Ochsner LSU Health Shreveport, Shreveport, LA; Ochsner LSU Health Shreveport, Shreveport, LA; LSU Health Shreveport, Shreveport, Louisiana

## Abstract

**Background:**

Tigecycline, a glycylcycline antibiotic, is commonly used against multi-drug resistant (MDR) Gram-negative (GN) bacteria. However, data on the hepatic safety of high-dose tigecycline (HDTIG) remains limited. This study describes the clinical features and outcomes of pts treated with HDTIG- versus standard dose- (SDTIG) based antibiotic regimen for MDR bacterial infections.

**Table 1.**
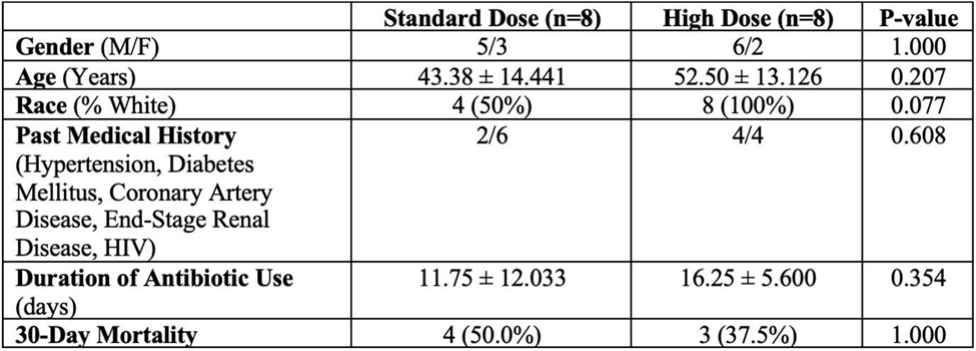
Baseline Characteristics of Study Population between Standard dose and High Dose Tigecycline groups

**Table 2.**
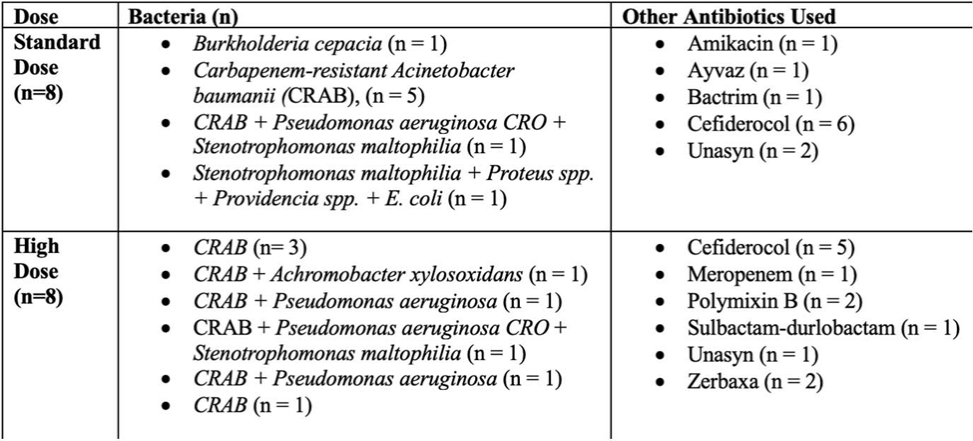
Bacterial Pathogens and Concomitant Antibiotics Used with Standard- vs High-Dose Tigecycline for MDR Gram-Negative bacterial Infections

**Methods:**

We conducted a retrospective study of adult pts treated with TIG for MDR-GN bacterial infections at Ochsner LSU Health–Academic Medical Center, between Jan 2019 and Feb 2025. Demographics, clinical features, microbiologic, treatment, and liver function data (AST, ALT, ALP, bilirubin) were collected at baseline and at the time of antibiotic therapy completion. Pts without liver function tests within 7 days of TIG initiation or discontinuation, pts treated with TIG < 48 hrs, or Pts treated for non-MDR-GN infections were all excluded. Statistical analyses were performed using Fisher’s exact test and paired and independent samples t-tests.

**Table 3.**
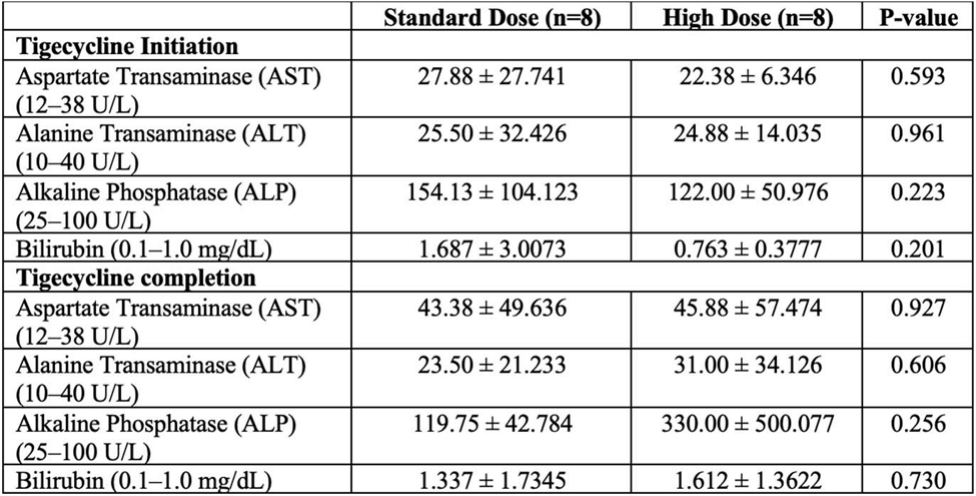
Liver Enzyme Values at Baseline Prior to Tigecycline Initiation and at the time of Tigecycline Completion

**Results:**

Sixteen pts were included and divided into groups [8 Pts HDTIG (100 mg every 12h) and 8 SDTIG (50 mg every 12h)]. The features are outlined in Tables 1, 2, and 3. HDTIG pts were more male (75.0% vs 62.5%) and White (100% vs 50%, p=0.07). No significant difference in age or medical comorbidities was observed. Infection sources in the SDTIG group were pneumonia (n=4), bacteremia (n=3), osteomyelitis (n=1), and SSTI (n=1); in the HDTIG group, pneumonia (n=4), SSTI (n=2), bacteremia (n=1), osteomyelitis (n=1), and UTI (n=1). CRAB was the most common pathogen in both groups. Only 1 pt in the SDTIG had an underlying liver disease. Baseline and post-treatment liver tests showed no significant differences between the two groups. ALP was numerically higher post-treatment in HDTIG (330.0 vs 119.8 U/L, p=0.256). The therapy of TIG duration was longer in HDTIG (16.3 vs 11.8 days, p=0.354) and the 30-day mortality was lower in HDTIG (37.5% vs 50.0%, p=1.0).

**Conclusion:**

Although the sample size was small in this cohort, HDTIG did not increase hepatotoxicity compared to SDTIG. The trends of ALP elevation in HDTIG warrant further investigation. Larger studies are needed to evaluate further the safety of HDTIG in Pts with MDR-GN infections.

**Disclosures:**

All Authors: No reported disclosures

